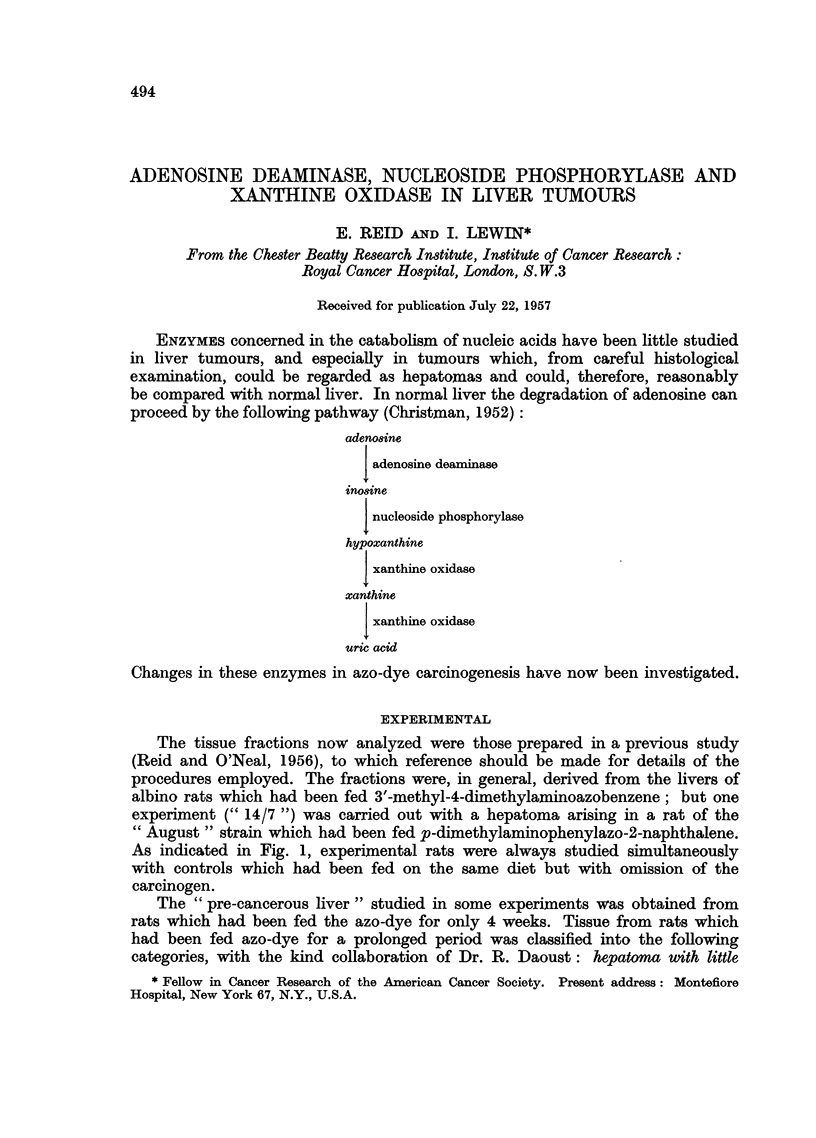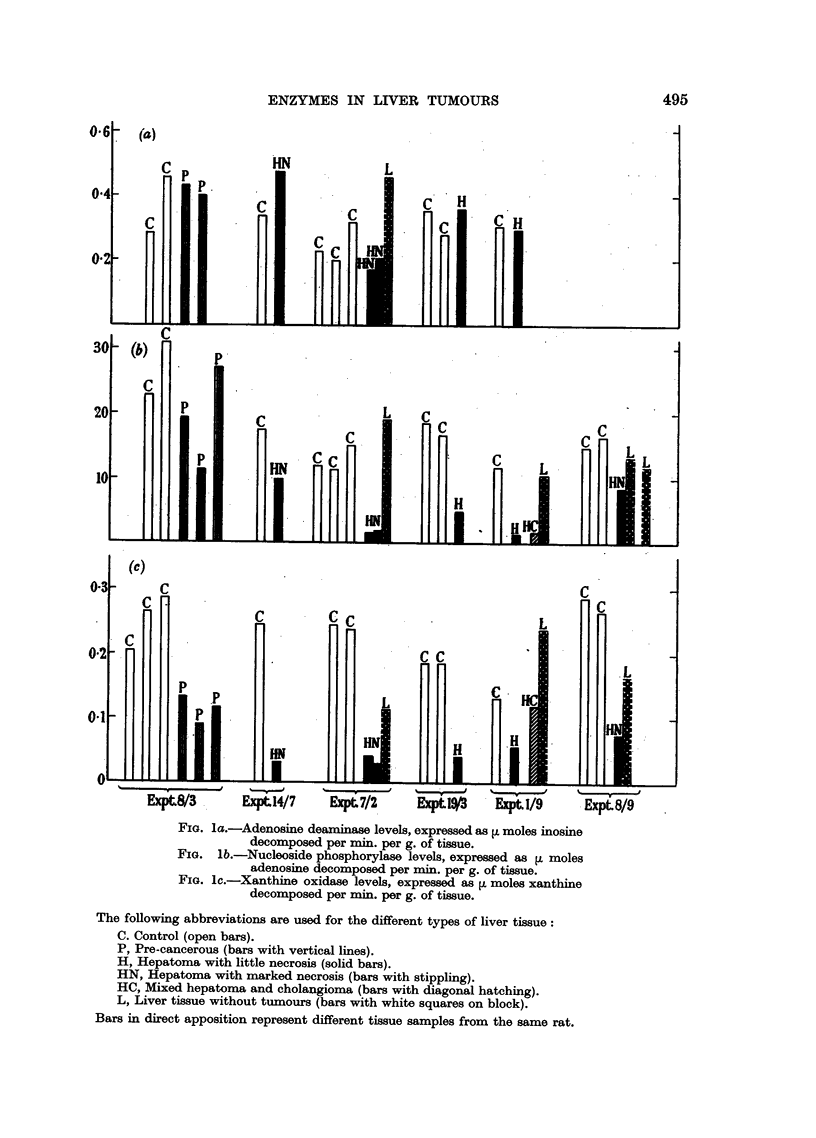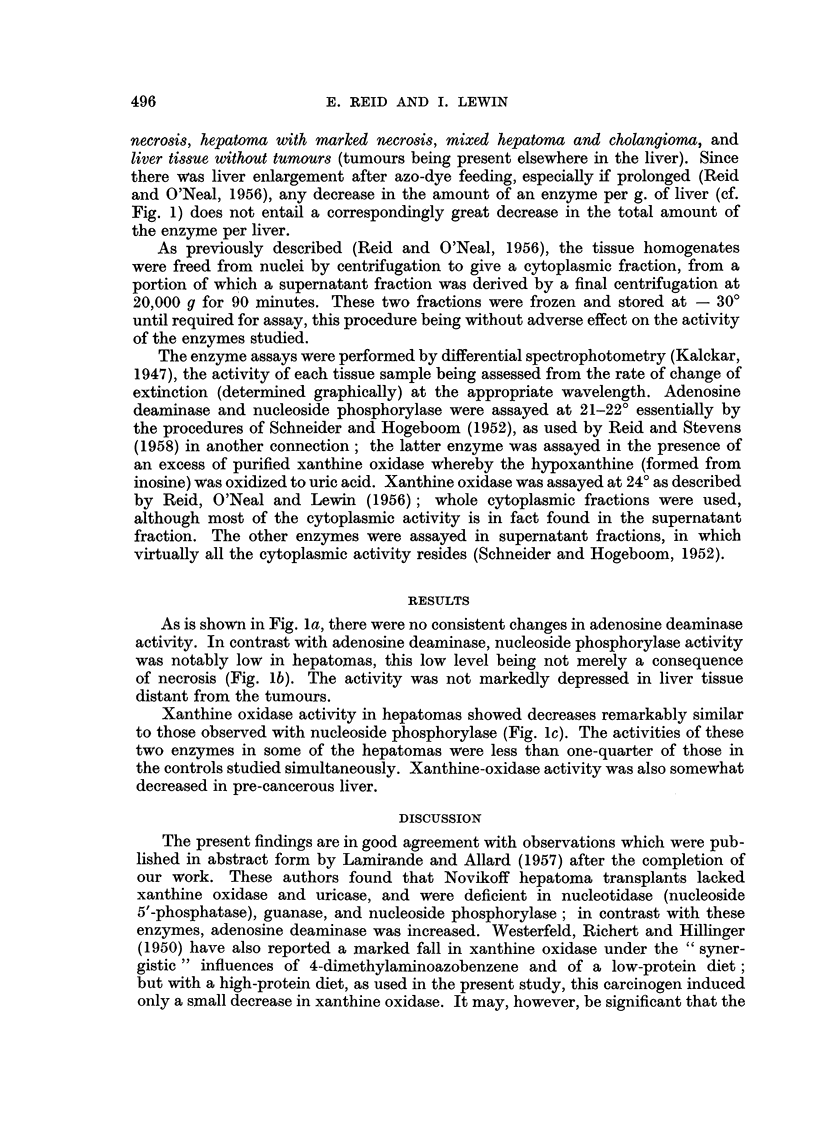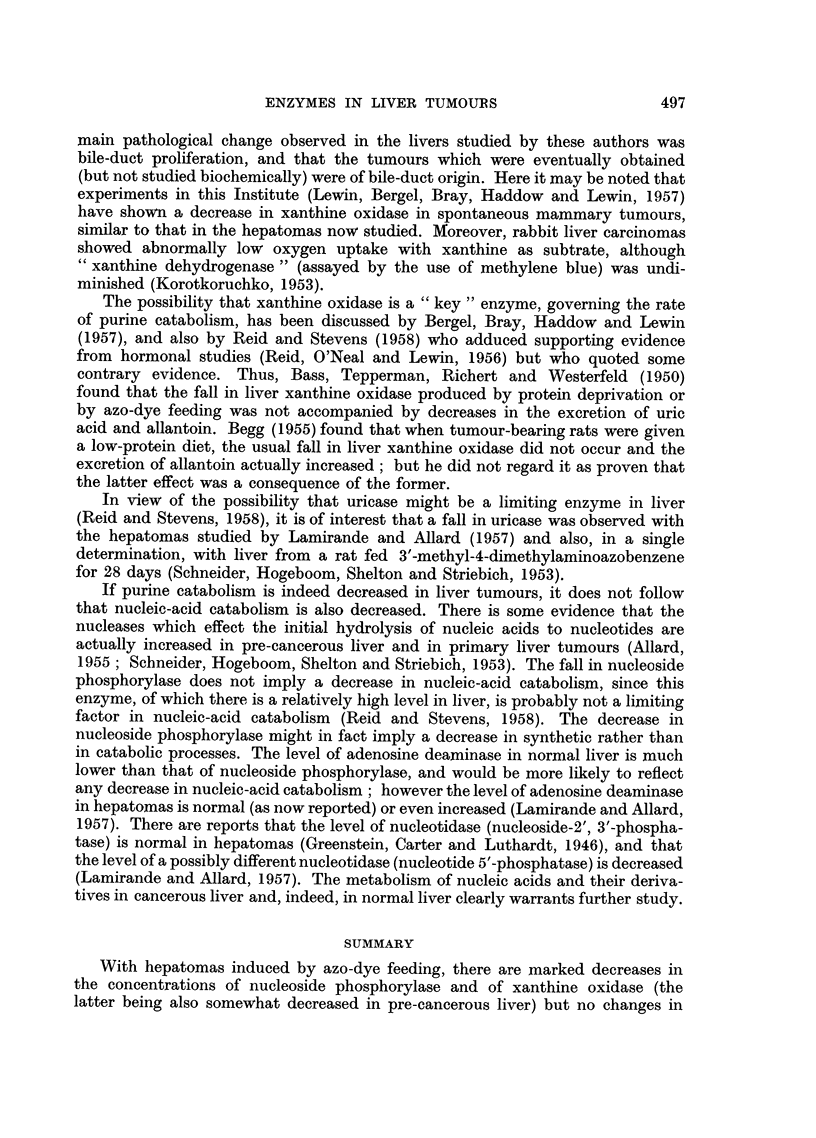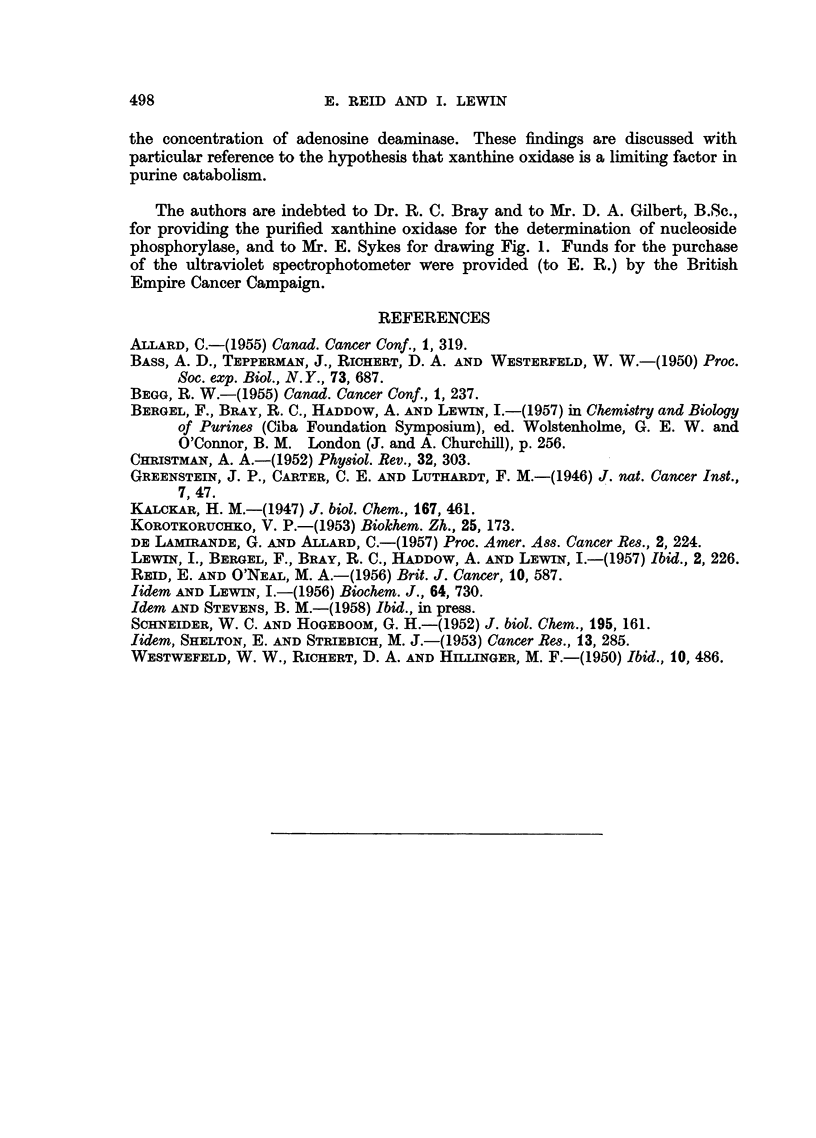# Adenosine Deaminase, Nucleoside Phosphorylase and Xanthine Oxidase in Liver Tumours

**DOI:** 10.1038/bjc.1957.60

**Published:** 1957-09

**Authors:** E. Reid, I. Lewin


### ADENOSINE DEAMINASE, NUCLEOSIDE PHOSPHORYLASE AND XANTHINE OXIDASE IN LIVER TUMOURS

E. REID AND I. LEWIN*

*From the Chester Beatty Research Institute, Institute of Cancer Research : Royal Cancer Hospital, London, S.W.3*

Received for publication July 22, 1957

ENZYMES concerned in the catabolism of nucleic acids have been little studied in liver tumours, and especially in tumours which, from careful histological examination, could be regarded as hepatomas and could, therefore, reasonably be compared with normal liver. In normal liver the degradation of adenosine can proceed by the following pathway (Christman, 1952) :

*adenosine*
↓ adenosine deaminase
*inosine*
↓ nucleoside phosphorylase
*hypoxanthine*
↓ xanthine oxidase
*xanthine*
↓ xanthine oxidase
*uric acid*

Changes in these enzymes in azo-dye carcinogenesis have now been investigated.

### EXPERIMENTAL

The tissue fractions now analyzed were those prepared in a previous study (Reid and O'Neal, 1956), to which reference should be made for details of the procedures employed. The fractions were, in general, derived from the livers of albino rats which had been fed 3'-methyl-4-dimethylaminoazobenzene ; but one experiment (" 14/7 ") was carried out with a hepatoma arising in a rat of the " August " strain which had been fed *p*-dimethylaminophenylazo-2-naphthalene. As indicated in Fig. 1, experimental rats were always studied simultaneously with controls which had been fed on the same diet but with omission of the carcinogen.

The " pre-cancerous liver " studied in some experiments was obtained from rats which had been fed the azo-dye for only 4 weeks. Tissue from rats which had been fed azo-dye for a prolonged period was classified into the following categories, with the kind collaboration of Dr. R. Daoust : *hepatoma with little*

* Fellow in Cancer Research of the American Cancer Society. Present address : Montefiore Hospital, New York 67, N.Y., U.S.A.

FIG. 1*a*.—Adenosine deaminase levels, expressed as $\mu$ moles inosine decomposed per min. per g. of tissue.

FIG. 1*b*.—Nucleoside phosphorylase levels, expressed as $\mu$ moles adenosine decomposed per min. per g. of tissue.

FIG. 1*c*.—Xanthine oxidase levels, expressed as $\mu$ moles xanthine decomposed per min. per g. of tissue.

The following abbreviations are used for the different types of liver tissue :

C. Control (open bars).
P, Pre-cancerous (bars with vertical lines).
H, Hepatoma with little necrosis (solid bars).
HN, Hepatoma with marked necrosis (bars with stippling).
HC, Mixed hepatoma and cholangioma (bars with diagonal hatching).
L, Liver tissue without tumours (bars with white squares on block).

Bars in direct apposition represent different tissue samples from the same rat.

*necrosis, hepatoma with marked necrosis, mixed hepatoma and cholangioma*, and *liver tissue without tumours* (tumours being present elsewhere in the liver). Since there was liver enlargement after azo-dye feeding, especially if prolonged (Reid and O'Neal, 1956), any decrease in the amount of an enzyme per g. of liver (cf. Fig. 1) does not entail a correspondingly great decrease in the total amount of the enzyme per liver.

As previously described (Reid and O'Neal, 1956), the tissue homogenates were freed from nuclei by centrifugation to give a cytoplasmic fraction, from a portion of which a supernatant fraction was derived by a final centrifugation at 20,000 *g* for 90 minutes. These two fractions were frozen and stored at — 30° until required for assay, this procedure being without adverse effect on the activity of the enzymes studied.

The enzyme assays were performed by differential spectrophotometry (Kalckar, 1947), the activity of each tissue sample being assessed from the rate of change of extinction (determined graphically) at the appropriate wavelength. Adenosine deaminase and nucleoside phosphorylase were assayed at 21–22° essentially by the procedures of Schneider and Hogeboom (1952), as used by Reid and Stevens (1958) in another connection ; the latter enzyme was assayed in the presence of an excess of purified xanthine oxidase whereby the hypoxanthine (formed from inosine) was oxidized to uric acid. Xanthine oxidase was assayed at 24° as described by Reid, O'Neal and Lewin (1956) ; whole cytoplasmic fractions were used, although most of the cytoplasmic activity is in fact found in the supernatant fraction. The other enzymes were assayed in supernatant fractions, in which virtually all the cytoplasmic activity resides (Schneider and Hogeboom, 1952).

### RESULTS

As is shown in Fig. 1*a*, there were no consistent changes in adenosine deaminase activity. In contrast with adenosine deaminase, nucleoside phosphorylase activity was notably low in hepatomas, this low level being not merely a consequence of necrosis (Fig. 1*b*). The activity was not markedly depressed in liver tissue distant from the tumours.

Xanthine oxidase activity in hepatomas showed decreases remarkably similar to those observed with nucleoside phosphorylase (Fig. 1*c*). The activities of these two enzymes in some of the hepatomas were less than one-quarter of those in the controls studied simultaneously. Xanthine-oxidase activity was also somewhat decreased in pre-cancerous liver.

### DISCUSSION

The present findings are in good agreement with observations which were published in abstract form by Lamirande and Allard (1957) after the completion of our work. These authors found that Novikoff hepatoma transplants lacked xanthine oxidase and uricase, and were deficient in nucleotidase (nucleoside 5′-phosphatase), guanase, and nucleoside phosphorylase ; in contrast with these enzymes, adenosine deaminase was increased. Westerfeld, Richert and Hillinger (1950) have also reported a marked fall in xanthine oxidase under the " synergistic " influences of 4-dimethylaminoazobenzene and of a low-protein diet ; but with a high-protein diet, as used in the present study, this carcinogen induced only a small decrease in xanthine oxidase. It may, however, be significant that the main pathological change observed in the livers studied by these authors was bile-duct proliferation, and that the tumours which were eventually obtained (but not studied biochemically) were of bile-duct origin. Here it may be noted that experiments in this Institute (Lewin, Bergel, Bray, Haddow and Lewin, 1957) have shown a decrease in xanthine oxidase in spontaneous mammary tumours, similar to that in the hepatomas now studied. Moreover, rabbit liver carcinomas showed abnormally low oxygen uptake with xanthine as subtrate, although " xanthine dehydrogenase " (assayed by the use of methylene blue) was undiminished (Korotkoruchko, 1953).

The possibility that xanthine oxidase is a " key " enzyme, governing the rate of purine catabolism, has been discussed by Bergel, Bray, Haddow and Lewin (1957), and also by Reid and Stevens (1958) who adduced supporting evidence from hormonal studies (Reid, O'Neal and Lewin, 1956) but who quoted some contrary evidence. Thus, Bass, Tepperman, Richert and Westerfeld (1950) found that the fall in liver xanthine oxidase produced by protein deprivation or by azo-dye feeding was not accompanied by decreases in the excretion of uric acid and allantoin. Begg (1955) found that when tumour-bearing rats were given a low-protein diet, the usual fall in liver xanthine oxidase did not occur and the excretion of allantoin actually increased ; but he did not regard it as proven that the latter effect was a consequence of the former.

In view of the possibility that uricase might be a limiting enzyme in liver (Reid and Stevens, 1958), it is of interest that a fall in uricase was observed with the hepatomas studied by Lamirande and Allard (1957) and also, in a single determination, with liver from a rat fed 3'-methyl-4-dimethylaminoazobenzene for 28 days (Schneider, Hogeboom, Shelton and Striebich, 1953).

If purine catabolism is indeed decreased in liver tumours, it does not follow that nucleic-acid catabolism is also decreased. There is some evidence that the nucleases which effect the initial hydrolysis of nucleic acids to nucleotides are actually increased in pre-cancerous liver and in primary liver tumours (Allard, 1955 ; Schneider, Hogeboom, Shelton and Striebich, 1953). The fall in nucleoside phosphorylase does not imply a decrease in nucleic-acid catabolism, since this enzyme, of which there is a relatively high level in liver, is probably not a limiting factor in nucleic-acid catabolism (Reid and Stevens, 1958). The decrease in nucleoside phosphorylase might in fact imply a decrease in synthetic rather than in catabolic processes. The level of adenosine deaminase in normal liver is much lower than that of nucleoside phosphorylase, and would be more likely to reflect any decrease in nucleic-acid catabolism ; however the level of adenosine deaminase in hepatomas is normal (as now reported) or even increased (Lamirande and Allard, 1957). There are reports that the level of nucleotidase (nucleoside-2', 3'-phosphatase) is normal in hepatomas (Greenstein, Carter and Luthardt, 1946), and that the level of a possibly different nucleotidase (nucleotide 5'-phosphatase) is decreased (Lamirande and Allard, 1957). The metabolism of nucleic acids and their derivatives in cancerous liver and, indeed, in normal liver clearly warrants further study.

### SUMMARY

With hepatomas induced by azo-dye feeding, there are marked decreases in the concentrations of nucleoside phosphorylase and of xanthine oxidase (the latter being also somewhat decreased in pre-cancerous liver) but no changes in the concentration of adenosine deaminase. These findings are discussed with particular reference to the hypothesis that xanthine oxidase is a limiting factor in purine catabolism.

The authors are indebted to Dr. R. C. Bray and to Mr. D. A. Gilbert, B.Sc., for providing the purified xanthine oxidase for the determination of nucleoside phosphorylase, and to Mr. E. Sykes for drawing Fig. 1. Funds for the purchase of the ultraviolet spectrophotometer were provided (to E. R.) by the British Empire Cancer Campaign.

### REFERENCES

ALLARD, C.—(1955) *Canad. Cancer Conf.*, **1**, 319.
BASS, A. D., TEPPERMAN, J., RICHERT, D. A. AND WESTERFELD, W. W.—(1950) *Proc. Soc. exp. Biol., N.Y.*, **73**, 687.
BEGG, R. W.—(1955) *Canad. Cancer Conf.*, **1**, 237.
BERGEL, F., BRAY, R. C., HADDOW, A. AND LEWIN, I.—(1957) in *Chemistry and Biology of Purines* (Ciba Foundation Symposium), ed. Wolstenholme, G. E. W. and O'Connor, B. M. London (J. and A. Churchill), p. 256.
CHRISTMAN, A. A.—(1952) *Physiol. Rev.*, **32**, 303.
GREENSTEIN, J. P., CARTER, C. E. AND LUTHARDT, F. M.—(1946) *J. nat. Cancer Inst.*, **7**, 47.
KALCKAR, H. M.—(1947) *J. biol. Chem.*, **167**, 461.
KOROTKORUCHKO, V. P.—(1953) *Biokhem. Zh.*, **25**, 173.
DE LAMIRANDE, G. AND ALLARD, C.—(1957) *Proc. Amer. Ass. Cancer Res.*, **2**, 224.
LEWIN, I., BERGEL, F., BRAY, R. C., HADDOW, A. AND LEWIN, I.—(1957) *Ibid.*, **2**, 226.
REID, E. AND O'NEAL, M. A.—(1956) *Brit. J. Cancer*, **10**, 587.
*Iidem* AND LEWIN, I.—(1956) *Biochem. J.*, **64**, 730.
*Idem* AND STEVENS, B. M.—(1958) *Ibid.*, in press.
SCHNEIDER, W. C. AND HOGEBOOM, G. H.—(1952) *J. biol. Chem.*, **195**, 161.
*Iidem*, SHELTON, E. AND STRIEBICH, M. J.—(1953) *Cancer Res.*, **13**, 285.
WESTWEFELD, W. W., RICHERT, D. A. AND HILLINGER, M. F.—(1950) *Ibid.*, **10**, 486.